# Poly[(μ_3_-benzene-1,3-dicarboxyl­ato-κ^4^ *O* ^1^:*O* ^1′^:*O* ^3^,*O* ^3′^)bis­(pyridine-κ*N*)cobalt(II)]

**DOI:** 10.1107/S1600536812020028

**Published:** 2012-05-12

**Authors:** Fengxia Xie, Dan Zhang, Xinxin Zhang

**Affiliations:** a School of Environment and Chemical Engineering, Xi’an Polytechnic University, Xi’an 710048, Shaanxi Province, People’s Republic of China

## Abstract

In the asymmetric unit of the title polymeric compound, [Co(C_8_H_4_O_4_)(C_5_H_5_N)_2_]_*n*_, there are two crystallographically independent Co^II^ atoms, each of which is six-coordinated in a distorted octa­hedral geometry by four O atoms from benzene­dicarboxyl­ate anions and two N atoms from pyridine ligands. The benzene­dicarboxyl­ate dianions bridge the Co^II^ atoms into a tape running along the *b* axis. C—H⋯O hydrogen bonds are observed in the tape and between the tapes.

## Related literature

For the synthesis and related structures, see: Abourahma *et al.* (2003 ▶). For compounds with metal-organic framework structures, see: Yan *et al.* (1996 ▶); Rosi *et al.* (2003 ▶); Jung *et al.* (2000 ▶); Chen *et al.* (2010 ▶).
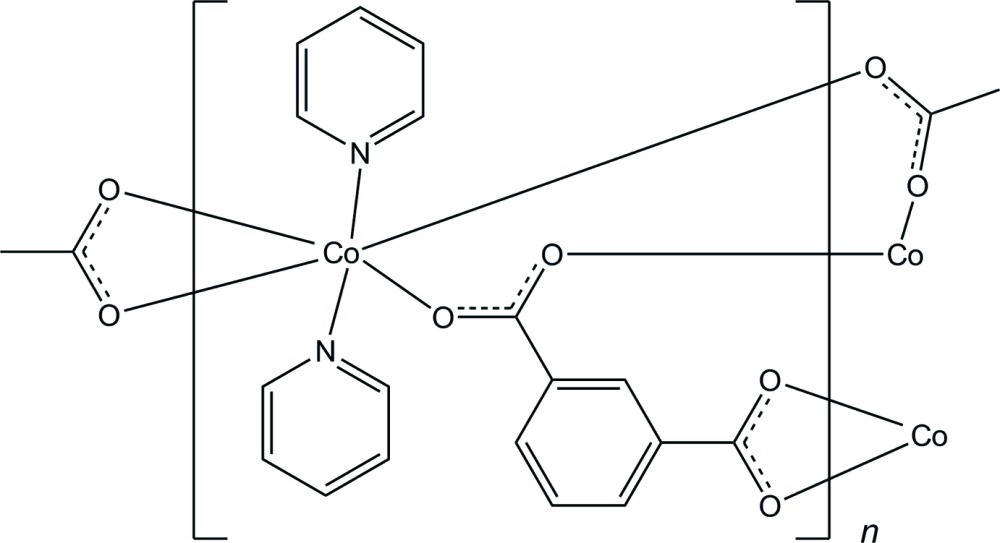


## Experimental

### 

#### Crystal data

[Co(C_8_H_4_O_4_)(C_5_H_5_N)_2_]
*M* *_r_* = 381.24
Monoclinic, 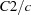

*a* = 26.7189 (18) Å
*b* = 10.1226 (7) Å
*c* = 24.7622 (17) Å
β = 96.421 (1)°
*V* = 6655.3 (8) Å^3^
*Z* = 16
Mo *K*α radiation
μ = 1.06 mm^−1^
*T* = 294 K
0.30 × 0.25 × 0.18 mm

#### Data collection

Bruker SMART APEX CCD area-detector diffractometer
Absorption correction: multi-scan (*SADABS*; Sheldrick, 1996 ▶) *T*_min_ = 0.742, *T*_max_ = 0.833
18265 measured reflections
5862 independent reflections
4733 reflections with *I* > 2σ(*I*)
*R*_int_ = 0.043

#### Refinement

*R*[*F* ^2^ > 2σ(*F* ^2^)] = 0.059
*wR*(*F* ^2^) = 0.142
*S* = 1.08
5862 reflections
451 parameters
H-atom parameters constrained
Δρ_max_ = 0.61 e Å^−3^
Δρ_min_ = −0.32 e Å^−3^

### 

Data collection: *SMART* (Bruker, 2003 ▶); cell refinement: *SAINT* (Bruker, 2003 ▶); data reduction: *SAINT*; program(s) used to solve structure: *SHELXS97* (Sheldrick, 2008 ▶); program(s) used to refine structure: *SHELXL97* (Sheldrick, 2008 ▶); molecular graphics: *ORTEP-3* (Farrugia, 1997 ▶); software used to prepare material for publication: *SHELXL97*.

## Supplementary Material

Crystal structure: contains datablock(s) I, global. DOI: 10.1107/S1600536812020028/is5111sup1.cif

Structure factors: contains datablock(s) I. DOI: 10.1107/S1600536812020028/is5111Isup2.hkl

Additional supplementary materials:  crystallographic information; 3D view; checkCIF report

## Figures and Tables

**Table 1:** Hydrogen-bond geometry (Å, °)

*D*—H⋯*A*	*D*—H	H⋯*A*	*D*⋯*A*	*D*—H⋯*A*
C3—H3⋯O8^i^	0.93	2.58	3.494 (5)	167
C11—H11⋯O4^ii^	0.93	2.54	3.461 (5)	171
C19—H19⋯O8^iii^	0.93	2.60	3.348 (7)	138
C23—H23⋯O3^iv^	0.93	2.53	3.213 (5)	130
C28—H28⋯O7^v^	0.93	2.55	3.313 (6)	139

Symmetry codes: (i) 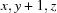; (ii) 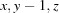; (iii) 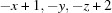; (iv) 
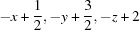
; (v) 
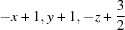
.

**Fig. 1.**
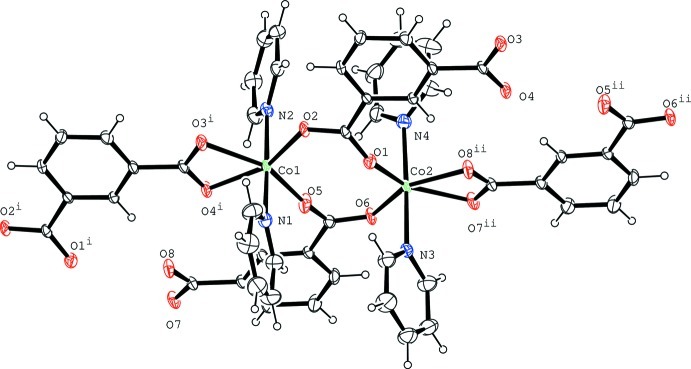
A part of the polymeric structure of the title compound. Displacement ellipsoids are drawn at the 25% probability level. [Symmetry codes: (i) x, y - 1, z; (ii) x, y + 1, z.]

**Fig. 2.**
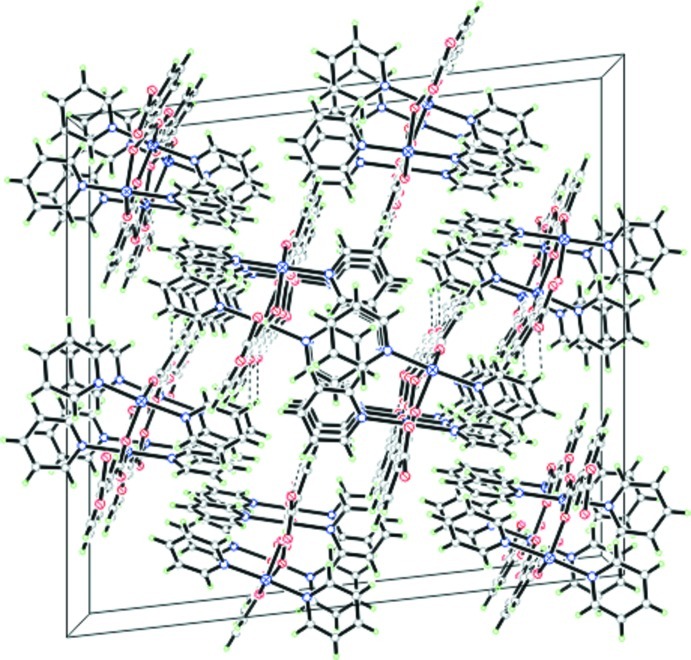
A packing diagram of the title compound, viewed down the b axis.

## supplementary crystallographic information

### Comment

During the last two decades, metal-organic frameworks (MOFs) have been developed rapidly due to their versatile structural diversities and their extensive potential applications in gas adsorptions, catalysis, magnetism and photochemistry (Rosi *et al.*, 2003; Jung *et al.*, 2000; Yan *et al.*, 1996; Chen *et al.*, 2010). Multi-carboxyl ligands, such as pyridine-2,5-dicarboxylic acid, isophthalic acid, 1,3,5-benzenetricarboxylic acid and 1,3,5-cyclohexanetricarboxylic acid, act as a three-connected node in conjunction with transition metals or lanthanide metals. These metals act as a six-connected node and then construct a 3,6-connecting rutile net.

In the crystal, the title cobalt(II) complex forms chains running along the *b* axis. As shown in Fig. 1, the coordination geometry around the Co(II) atom is a slightly distorted octahedron. In the octahedron unit, the axial positions are occupied by N atoms from pyridine ligands and the equatorial sites by O atoms from the 1,3-benzenedicarboxylate dianions. The octahedral coordination sphere of the cobalt(II) cation is slightly distorted with distances in the range of 2.019 (3) to 2.302 (3) Å.

### Experimental

Purple crystals of the title compound were obtained from slow diffusion of ethanol(6 mL) of 1,3-H_2_bdc (83.0 mg, 0.5 mmol) and pyridine (0.15 ml) into an aqueous solution (3 ml) of cobalt chloride (119.4 mg, 0.5 mmol).

### Refinement

All H atoms were located in difference maps and then treated as riding atoms in geometrically idealized positions (C—H = 0.93 Å), with *U*_iso_(H) = 1.2*U*_eq_(C).

### Crystal data

**Table:**

[Co(C_8_H_4_O_4_)(C_5_H_5_N)_2_]	*F*(000) = 3120
*M**_r_* = 381.24	*D*_x_ = 1.522 Mg m^−^^3^
Monoclinic, *C*2/*c*	Mo *K*α radiation, λ = 0.71073 Å
Hall symbol: -C 2yc	Cell parameters from 2813 reflections
*a* = 26.7189 (18) Å	θ = 2.3–23.0°
*b* = 10.1226 (7) Å	µ = 1.06 mm^−^^1^
*c* = 24.7622 (17) Å	*T* = 294 K
β = 96.421 (1)°	Block, purple
*V* = 6655.3 (8) Å^3^	0.30 × 0.25 × 0.18 mm
*Z* = 16	

### Data collection

**Table:**

Bruker SMART APEX CCD area-detector diffractometer	5862 independent reflections
Radiation source: fine-focus sealed tube	4733 reflections with *I* > 2σ(*I*)
Graphite monochromator	*R*_int_ = 0.043
φ and ω scans	θ_max_ = 25.1°, θ_min_ = 1.5°
Absorption correction: multi-scan (*SADABS*; Sheldrick, 1996)	*h* = −31→31
*T*_min_ = 0.742, *T*_max_ = 0.833	*k* = −10→12
18265 measured reflections	*l* = −26→29

### Refinement

**Table:**

Refinement on *F*^2^	Primary atom site location: structure-invariant direct methods
Least-squares matrix: full	Secondary atom site location: difference Fourier map
*R*[*F*^2^ > 2σ(*F*^2^)] = 0.059	Hydrogen site location: inferred from neighbouring sites
*wR*(*F*^2^) = 0.142	H-atom parameters constrained
*S* = 1.08	*w* = 1/[σ^2^(*F*_o_^2^) + (0.0766*P*)^2^ + 0.0224*P*] where *P* = (*F*_o_^2^ + 2*F*_c_^2^)/3
5862 reflections	(Δ/σ)_max_ = 0.001
451 parameters	Δρ_max_ = 0.61 e Å^−^^3^
0 restraints	Δρ_min_ = −0.32 e Å^−^^3^

### Special details

**Table:**

Geometry. All e.s.d.'s (except the e.s.d. in the dihedral angle between two l.s. planes) are estimated using the full covariance matrix. The cell e.s.d.'s are taken into account individually in the estimation of e.s.d.'s in distances, angles and torsion angles; correlations between e.s.d.'s in cell parameters are only used when they are defined by crystal symmetry. An approximate (isotropic) treatment of cell e.s.d.'s is used for estimating e.s.d.'s involving l.s. planes.
Refinement. Refinement of *F*^2^ against ALL reflections. The weighted *R*-factor *wR* and goodness of fit *S* are based on *F*^2^, conventional *R*-factors *R* are based on *F*, with *F* set to zero for negative *F*^2^. The threshold expression of *F*^2^ > σ(*F*^2^) is used only for calculating *R*-factors(gt) *etc*. and is not relevant to the choice of reflections for refinement. *R*-factors based on *F*^2^ are statistically about twice as large as those based on *F*, and *R*- factors based on ALL data will be even larger.

### Fractional atomic coordinates and isotropic or equivalent isotropic displacement parameters (Å^2^)

**Table:**

	*x*	*y*	*z*	*U*_iso_*/*U*_eq_	
Co1	0.35527 (2)	0.18383 (5)	0.93942 (2)	0.03237 (18)	
Co2	0.39249 (2)	0.51173 (5)	0.84317 (2)	0.02943 (17)	
O4	0.35636 (11)	0.9743 (3)	0.93314 (11)	0.0402 (7)	
O3	0.32957 (10)	1.0611 (3)	1.00560 (11)	0.0392 (7)	
O6	0.39712 (11)	0.3391 (2)	0.79982 (11)	0.0388 (7)	
O8	0.39911 (11)	−0.2787 (3)	0.84887 (11)	0.0429 (7)	
O7	0.40607 (11)	−0.3629 (3)	0.76863 (12)	0.0433 (7)	
O5	0.38365 (13)	0.2080 (3)	0.86745 (12)	0.0542 (9)	
O1	0.38119 (11)	0.4917 (3)	0.92200 (11)	0.0390 (7)	
O2	0.34045 (10)	0.3598 (2)	0.97351 (12)	0.0395 (7)	
N2	0.27837 (13)	0.1840 (3)	0.89985 (13)	0.0381 (8)	
N1	0.43006 (13)	0.1840 (3)	0.98289 (15)	0.0437 (9)	
N4	0.31207 (13)	0.5260 (4)	0.82074 (14)	0.0426 (9)	
N3	0.47206 (12)	0.4904 (3)	0.86490 (13)	0.0352 (8)	
C29	0.57532 (19)	0.4548 (6)	0.8862 (2)	0.0676 (16)	
H29	0.6099	0.4427	0.8935	0.081*	
C22	0.24213 (16)	0.2616 (4)	0.91632 (17)	0.0419 (10)	
H22	0.2506	0.3179	0.9456	0.050*	
C23	0.19321 (17)	0.2620 (5)	0.89223 (18)	0.0487 (12)	
H23	0.1695	0.3181	0.9048	0.058*	
C25	0.21609 (19)	0.0967 (5)	0.83247 (19)	0.0544 (13)	
H25	0.2080	0.0381	0.8039	0.065*	
C26	0.26455 (18)	0.1030 (5)	0.85853 (18)	0.0486 (12)	
H26	0.2888	0.0476	0.8465	0.058*	
C36	0.28471 (17)	0.4256 (5)	0.79786 (19)	0.0524 (12)	
H36	0.3013	0.3471	0.7920	0.063*	
C24	0.17994 (18)	0.1788 (5)	0.8495 (2)	0.0536 (13)	
H24	0.1472	0.1777	0.8324	0.064*	
C32	0.28686 (19)	0.6354 (6)	0.8290 (2)	0.0608 (14)	
H32	0.3047	0.7069	0.8449	0.073*	
C34	0.2091 (2)	0.5456 (7)	0.7917 (2)	0.0711 (17)	
H34	0.1746	0.5526	0.7820	0.085*	
C17	0.43602 (19)	0.2021 (5)	1.0365 (2)	0.0618 (14)	
H17	0.4074	0.2191	1.0535	0.074*	
C21	0.47211 (19)	0.1616 (5)	0.9598 (2)	0.0589 (14)	
H21	0.4695	0.1499	0.9223	0.071*	
C18	0.4813 (2)	0.1976 (6)	1.0685 (2)	0.0733 (17)	
H18	0.4832	0.2105	1.1058	0.088*	
C33	0.2356 (2)	0.6492 (7)	0.8152 (2)	0.0787 (19)	
H33	0.2195	0.7280	0.8219	0.094*	
C19	0.5237 (2)	0.1736 (6)	1.0439 (3)	0.0759 (18)	
H19	0.5550	0.1700	1.0644	0.091*	
C35	0.23382 (19)	0.4310 (6)	0.7825 (2)	0.0670 (16)	
H35	0.2166	0.3588	0.7664	0.080*	
C20	0.5197 (2)	0.1550 (6)	0.9892 (3)	0.0762 (18)	
H20	0.5481	0.1381	0.9718	0.091*	
C10	0.41050 (13)	0.1095 (4)	0.78952 (15)	0.0283 (8)	
C12	0.41443 (14)	−0.1281 (4)	0.77838 (15)	0.0299 (9)	
C9	0.39595 (14)	0.2272 (4)	0.82185 (16)	0.0300 (9)	
C11	0.40432 (14)	−0.0177 (3)	0.80902 (16)	0.0285 (9)	
H11	0.3933	−0.0292	0.8430	0.034*	
C4	0.32940 (14)	0.8249 (4)	0.99897 (15)	0.0286 (9)	
C1	0.35306 (13)	0.4719 (4)	0.95844 (15)	0.0265 (8)	
C7	0.29152 (15)	0.6799 (4)	1.05964 (16)	0.0370 (10)	
H7	0.2735	0.6675	1.0893	0.044*	
C16	0.42825 (16)	0.1257 (4)	0.73977 (17)	0.0396 (10)	
H16	0.4328	0.2104	0.7266	0.048*	
C2	0.33449 (13)	0.5891 (4)	0.98750 (15)	0.0250 (8)	
C13	0.40588 (14)	−0.2644 (4)	0.79965 (17)	0.0335 (9)	
C8	0.30677 (14)	0.5715 (4)	1.03103 (16)	0.0316 (9)	
H8	0.2983	0.4867	1.0412	0.038*	
C6	0.30321 (15)	0.8062 (4)	1.04375 (16)	0.0346 (9)	
H6	0.2935	0.8788	1.0631	0.041*	
C3	0.34509 (13)	0.7163 (3)	0.97101 (15)	0.0254 (8)	
H3	0.3628	0.7288	0.9411	0.030*	
C14	0.43154 (16)	−0.1094 (4)	0.72835 (17)	0.0427 (11)	
H14	0.4379	−0.1824	0.7073	0.051*	
C15	0.43943 (18)	0.0167 (4)	0.70894 (19)	0.0496 (12)	
H15	0.4520	0.0283	0.6757	0.059*	
C5	0.33932 (14)	0.9617 (4)	0.97830 (17)	0.0307 (9)	
C27	0.50228 (17)	0.4938 (5)	0.8263 (2)	0.0554 (13)	
H27	0.4879	0.5093	0.7909	0.067*	
C31	0.49385 (18)	0.4702 (5)	0.91487 (19)	0.0530 (13)	
H31	0.4736	0.4684	0.9431	0.064*	
C30	0.54507 (19)	0.4515 (6)	0.9272 (2)	0.0721 (17)	
H30	0.5588	0.4369	0.9629	0.087*	
C28	0.55390 (18)	0.4760 (6)	0.8348 (2)	0.0663 (16)	
H28	0.5734	0.4785	0.8060	0.080*	

### Atomic displacement parameters (Å^2^)

**Table:**

	*U*^11^	*U*^22^	*U*^33^	*U*^12^	*U*^13^	*U*^23^
Co1	0.0451 (3)	0.0139 (3)	0.0399 (3)	0.0011 (2)	0.0127 (3)	−0.0004 (2)
Co2	0.0387 (3)	0.0160 (3)	0.0339 (3)	0.0021 (2)	0.0057 (2)	−0.0004 (2)
O4	0.0606 (19)	0.0169 (15)	0.0462 (18)	0.0034 (13)	0.0202 (15)	0.0024 (12)
O3	0.0546 (18)	0.0159 (15)	0.0494 (18)	−0.0012 (13)	0.0154 (14)	−0.0031 (12)
O6	0.0606 (19)	0.0138 (15)	0.0427 (17)	0.0007 (13)	0.0092 (14)	0.0001 (12)
O8	0.072 (2)	0.0198 (15)	0.0373 (17)	−0.0019 (14)	0.0099 (15)	0.0027 (12)
O7	0.064 (2)	0.0174 (15)	0.0489 (18)	0.0039 (14)	0.0072 (15)	−0.0051 (13)
O5	0.093 (3)	0.0279 (17)	0.0487 (19)	−0.0023 (16)	0.0386 (18)	−0.0011 (14)
O1	0.0536 (18)	0.0273 (16)	0.0394 (17)	0.0028 (13)	0.0192 (14)	−0.0009 (12)
O2	0.0552 (18)	0.0103 (14)	0.0563 (19)	−0.0009 (12)	0.0202 (14)	−0.0029 (12)
N2	0.051 (2)	0.027 (2)	0.037 (2)	0.0022 (16)	0.0069 (16)	−0.0002 (15)
N1	0.044 (2)	0.030 (2)	0.057 (2)	−0.0025 (16)	0.0100 (18)	−0.0032 (17)
N4	0.044 (2)	0.046 (2)	0.038 (2)	0.0050 (18)	0.0057 (17)	0.0056 (17)
N3	0.041 (2)	0.0278 (19)	0.037 (2)	0.0022 (15)	0.0066 (16)	0.0014 (14)
C29	0.039 (3)	0.089 (5)	0.074 (4)	0.000 (3)	0.002 (3)	0.019 (3)
C22	0.056 (3)	0.031 (2)	0.041 (2)	0.000 (2)	0.015 (2)	−0.0032 (19)
C23	0.048 (3)	0.051 (3)	0.049 (3)	0.004 (2)	0.014 (2)	0.001 (2)
C25	0.070 (3)	0.049 (3)	0.044 (3)	−0.002 (3)	0.005 (2)	−0.010 (2)
C26	0.064 (3)	0.037 (3)	0.045 (3)	0.003 (2)	0.008 (2)	−0.006 (2)
C36	0.049 (3)	0.050 (3)	0.059 (3)	−0.006 (2)	0.005 (2)	0.017 (2)
C24	0.048 (3)	0.059 (4)	0.052 (3)	−0.006 (2)	−0.002 (2)	0.008 (2)
C32	0.056 (3)	0.071 (4)	0.053 (3)	0.020 (3)	−0.006 (2)	−0.015 (3)
C34	0.043 (3)	0.116 (6)	0.054 (3)	0.011 (3)	0.002 (3)	0.003 (3)
C17	0.049 (3)	0.069 (4)	0.068 (4)	−0.010 (3)	0.007 (3)	−0.017 (3)
C21	0.053 (3)	0.053 (3)	0.073 (4)	0.003 (3)	0.017 (3)	0.002 (3)
C18	0.069 (4)	0.080 (5)	0.068 (4)	−0.007 (3)	−0.007 (3)	−0.016 (3)
C33	0.065 (4)	0.112 (6)	0.058 (4)	0.037 (4)	0.002 (3)	−0.015 (3)
C19	0.054 (3)	0.067 (4)	0.102 (5)	−0.014 (3)	−0.013 (3)	−0.003 (4)
C35	0.049 (3)	0.080 (4)	0.071 (4)	−0.021 (3)	0.000 (3)	0.015 (3)
C20	0.045 (3)	0.077 (5)	0.108 (5)	0.004 (3)	0.017 (3)	0.002 (4)
C10	0.030 (2)	0.019 (2)	0.037 (2)	0.0010 (16)	0.0054 (17)	−0.0022 (16)
C12	0.035 (2)	0.020 (2)	0.035 (2)	0.0008 (17)	0.0040 (17)	−0.0029 (16)
C9	0.030 (2)	0.021 (2)	0.038 (2)	−0.0015 (16)	0.0028 (17)	−0.0032 (17)
C11	0.036 (2)	0.018 (2)	0.033 (2)	0.0034 (16)	0.0089 (17)	0.0002 (16)
C4	0.032 (2)	0.016 (2)	0.038 (2)	0.0018 (16)	0.0020 (17)	0.0015 (16)
C1	0.030 (2)	0.015 (2)	0.033 (2)	−0.0012 (15)	−0.0026 (17)	−0.0017 (15)
C7	0.047 (2)	0.028 (2)	0.039 (2)	−0.0022 (19)	0.0186 (19)	0.0029 (18)
C16	0.053 (3)	0.021 (2)	0.048 (3)	−0.0043 (19)	0.015 (2)	0.0039 (18)
C2	0.0246 (18)	0.0172 (19)	0.032 (2)	0.0039 (15)	0.0003 (15)	0.0012 (15)
C13	0.036 (2)	0.020 (2)	0.045 (3)	0.0055 (17)	0.0048 (18)	0.0055 (18)
C8	0.038 (2)	0.017 (2)	0.041 (2)	0.0020 (17)	0.0071 (18)	0.0079 (16)
C6	0.045 (2)	0.025 (2)	0.035 (2)	0.0054 (18)	0.0122 (18)	−0.0048 (17)
C3	0.0260 (19)	0.0180 (19)	0.032 (2)	−0.0002 (15)	0.0046 (16)	−0.0012 (15)
C14	0.056 (3)	0.031 (3)	0.044 (3)	−0.002 (2)	0.016 (2)	−0.0073 (19)
C15	0.074 (3)	0.032 (3)	0.048 (3)	−0.005 (2)	0.032 (2)	−0.005 (2)
C5	0.033 (2)	0.014 (2)	0.046 (2)	0.0025 (16)	0.0031 (18)	−0.0012 (16)
C27	0.048 (3)	0.078 (4)	0.041 (3)	0.005 (3)	0.007 (2)	0.004 (2)
C31	0.049 (3)	0.067 (4)	0.043 (3)	0.002 (2)	0.006 (2)	0.006 (2)
C30	0.054 (3)	0.103 (5)	0.054 (3)	−0.002 (3)	−0.015 (3)	0.018 (3)
C28	0.047 (3)	0.085 (4)	0.069 (4)	0.001 (3)	0.018 (3)	0.013 (3)

### Geometric parameters (Å, º)

**Table:**

Co1—O5	2.028 (3)	C32—H32	0.9300
Co1—O2	2.029 (3)	C34—C33	1.358 (8)
Co1—O4^i^	2.127 (3)	C34—C35	1.366 (8)
Co1—N1	2.161 (4)	C34—H34	0.9300
Co1—N2	2.175 (3)	C17—C18	1.371 (7)
Co1—O3^i^	2.225 (3)	C17—H17	0.9300
Co2—O1	2.019 (3)	C21—C20	1.394 (7)
Co2—O6	2.062 (3)	C21—H21	0.9300
Co2—O8^ii^	2.132 (3)	C18—C19	1.366 (8)
Co2—N3	2.145 (3)	C18—H18	0.9300
Co2—N4	2.163 (4)	C33—H33	0.9300
Co2—O7^ii^	2.302 (3)	C19—C20	1.360 (8)
O4—C5	1.260 (5)	C19—H19	0.9300
O4—Co1^ii^	2.127 (3)	C35—H35	0.9300
O3—C5	1.256 (4)	C20—H20	0.9300
O3—Co1^ii^	2.225 (3)	C10—C16	1.378 (5)
O6—C9	1.259 (4)	C10—C11	1.391 (5)
O8—C13	1.261 (5)	C10—C9	1.511 (5)
O8—Co2^i^	2.132 (3)	C12—C14	1.381 (5)
O7—C13	1.260 (5)	C12—C11	1.394 (5)
O7—Co2^i^	2.302 (3)	C12—C13	1.503 (5)
O5—C9	1.227 (5)	C11—H11	0.9300
O1—C1	1.253 (4)	C4—C3	1.389 (5)
O2—C1	1.252 (4)	C4—C6	1.389 (5)
N2—C26	1.331 (5)	C4—C5	1.510 (5)
N2—C22	1.345 (5)	C1—C2	1.500 (5)
N1—C17	1.332 (6)	C7—C6	1.384 (5)
N1—C21	1.336 (6)	C7—C8	1.391 (5)
N4—C32	1.324 (6)	C7—H7	0.9300
N4—C36	1.340 (6)	C16—C15	1.393 (6)
N3—C27	1.319 (5)	C16—H16	0.9300
N3—C31	1.323 (5)	C2—C8	1.386 (5)
C29—C28	1.352 (7)	C2—C3	1.390 (5)
C29—C30	1.368 (7)	C8—H8	0.9300
C29—H29	0.9300	C6—H6	0.9300
C22—C23	1.375 (6)	C3—H3	0.9300
C22—H22	0.9300	C14—C15	1.389 (6)
C23—C24	1.367 (6)	C14—H14	0.9300
C23—H23	0.9300	C15—H15	0.9300
C25—C24	1.376 (6)	C5—Co1^ii^	2.501 (4)
C25—C26	1.382 (6)	C27—C28	1.384 (6)
C25—H25	0.9300	C27—H27	0.9300
C26—H26	0.9300	C31—C30	1.382 (6)
C36—C35	1.371 (6)	C31—H31	0.9300
C36—H36	0.9300	C30—H30	0.9300
C24—H24	0.9300	C28—H28	0.9300
C32—C33	1.382 (7)		
			
O5—Co1—O2	111.67 (12)	C18—C17—H17	117.6
O5—Co1—O4^i^	92.66 (11)	N1—C21—C20	123.1 (5)
O2—Co1—O4^i^	155.61 (11)	N1—C21—H21	118.5
O5—Co1—N1	91.25 (14)	C20—C21—H21	118.5
O2—Co1—N1	90.28 (13)	C19—C18—C17	118.1 (6)
O4^i^—Co1—N1	91.02 (12)	C19—C18—H18	120.9
O5—Co1—N2	91.79 (13)	C17—C18—H18	120.9
O2—Co1—N2	88.19 (12)	C34—C33—C32	118.8 (6)
O4^i^—Co1—N2	89.29 (12)	C34—C33—H33	120.6
N1—Co1—N2	176.93 (13)	C32—C33—H33	120.6
O5—Co1—O3^i^	152.97 (11)	C20—C19—C18	119.4 (5)
O2—Co1—O3^i^	95.36 (10)	C20—C19—H19	120.3
O4^i^—Co1—O3^i^	60.33 (10)	C18—C19—H19	120.3
N1—Co1—O3^i^	88.73 (12)	C34—C35—C36	118.0 (6)
N2—Co1—O3^i^	88.77 (11)	C34—C35—H35	121.0
O1—Co2—O6	116.27 (11)	C36—C35—H35	121.0
O1—Co2—O8^ii^	93.21 (11)	C19—C20—C21	118.6 (5)
O6—Co2—O8^ii^	150.27 (11)	C19—C20—H20	120.7
O1—Co2—N3	89.92 (12)	C21—C20—H20	120.7
O6—Co2—N3	85.98 (12)	C16—C10—C11	119.1 (3)
O8^ii^—Co2—N3	90.61 (12)	C16—C10—C9	120.9 (3)
O1—Co2—N4	90.19 (12)	C11—C10—C9	119.9 (3)
O6—Co2—N4	92.22 (13)	C14—C12—C11	118.9 (4)
O8^ii^—Co2—N4	91.34 (13)	C14—C12—C13	121.2 (4)
N3—Co2—N4	178.04 (14)	C11—C12—C13	119.9 (3)
O1—Co2—O7^ii^	152.23 (10)	O5—C9—O6	124.3 (4)
O6—Co2—O7^ii^	91.47 (10)	O5—C9—C10	118.3 (3)
O8^ii^—Co2—O7^ii^	59.03 (10)	O6—C9—C10	117.4 (3)
N3—Co2—O7^ii^	90.91 (12)	C10—C11—C12	121.0 (4)
N4—Co2—O7^ii^	89.92 (12)	C10—C11—H11	119.5
C5—O4—Co1^ii^	91.5 (2)	C12—C11—H11	119.5
C5—O3—Co1^ii^	87.2 (2)	C3—C4—C6	119.8 (3)
C9—O6—Co2	122.1 (3)	C3—C4—C5	118.9 (3)
C13—O8—Co2^i^	94.0 (2)	C6—C4—C5	121.3 (3)
C13—O7—Co2^i^	86.3 (2)	O2—C1—O1	124.2 (3)
C9—O5—Co1	173.3 (3)	O2—C1—C2	117.3 (3)
C1—O1—Co2	151.5 (3)	O1—C1—C2	118.4 (3)
C1—O2—Co1	126.9 (3)	C6—C7—C8	119.7 (4)
C26—N2—C22	116.5 (4)	C6—C7—H7	120.1
C26—N2—Co1	120.9 (3)	C8—C7—H7	120.1
C22—N2—Co1	122.7 (3)	C10—C16—C15	120.8 (4)
C17—N1—C21	116.0 (4)	C10—C16—H16	119.6
C17—N1—Co1	119.8 (3)	C15—C16—H16	119.6
C21—N1—Co1	124.2 (3)	C8—C2—C3	119.4 (3)
C32—N4—C36	115.9 (4)	C8—C2—C1	120.4 (3)
C32—N4—Co2	121.6 (3)	C3—C2—C1	120.2 (3)
C36—N4—Co2	122.5 (3)	O7—C13—O8	120.6 (4)
C27—N3—C31	116.2 (4)	O7—C13—C12	120.2 (4)
C27—N3—Co2	119.0 (3)	O8—C13—C12	119.2 (4)
C31—N3—Co2	124.8 (3)	C2—C8—C7	120.5 (3)
C28—C29—C30	118.7 (5)	C2—C8—H8	119.8
C28—C29—H29	120.7	C7—C8—H8	119.8
C30—C29—H29	120.7	C7—C6—C4	120.2 (4)
N2—C22—C23	123.5 (4)	C7—C6—H6	119.9
N2—C22—H22	118.2	C4—C6—H6	119.9
C23—C22—H22	118.2	C4—C3—C2	120.3 (3)
C24—C23—C22	119.0 (4)	C4—C3—H3	119.9
C24—C23—H23	120.5	C2—C3—H3	119.9
C22—C23—H23	120.5	C12—C14—C15	121.0 (4)
C24—C25—C26	118.8 (4)	C12—C14—H14	119.5
C24—C25—H25	120.6	C15—C14—H14	119.5
C26—C25—H25	120.6	C14—C15—C16	119.2 (4)
N2—C26—C25	123.5 (4)	C14—C15—H15	120.4
N2—C26—H26	118.2	C16—C15—H15	120.4
C25—C26—H26	118.2	O3—C5—O4	120.9 (4)
N4—C36—C35	124.3 (5)	O3—C5—C4	119.8 (4)
N4—C36—H36	117.8	O4—C5—C4	119.2 (3)
C35—C36—H36	117.8	N3—C27—C28	124.5 (5)
C23—C24—C25	118.6 (5)	N3—C27—H27	117.7
C23—C24—H24	120.7	C28—C27—H27	117.7
C25—C24—H24	120.7	N3—C31—C30	123.4 (4)
N4—C32—C33	123.7 (5)	N3—C31—H31	118.3
N4—C32—H32	118.2	C30—C31—H31	118.3
C33—C32—H32	118.2	C29—C30—C31	119.0 (5)
C33—C34—C35	119.2 (5)	C29—C30—H30	120.5
C33—C34—H34	120.4	C31—C30—H30	120.5
C35—C34—H34	120.4	C29—C28—C27	118.3 (5)
N1—C17—C18	124.8 (5)	C29—C28—H28	120.9
N1—C17—H17	117.6	C27—C28—H28	120.9
			
O1—Co2—O6—C9	−4.0 (3)	N4—C32—C33—C34	−0.2 (9)
O8^ii^—Co2—O6—C9	168.1 (3)	C17—C18—C19—C20	−0.1 (9)
N3—Co2—O6—C9	83.9 (3)	C33—C34—C35—C36	0.3 (8)
N4—Co2—O6—C9	−95.3 (3)	N4—C36—C35—C34	−0.8 (8)
O7^ii^—Co2—O6—C9	174.7 (3)	C18—C19—C20—C21	0.2 (9)
O6—Co2—O1—C1	−80.0 (5)	N1—C21—C20—C19	−0.8 (9)
O8^ii^—Co2—O1—C1	103.9 (5)	Co2—O6—C9—O5	12.9 (6)
N3—Co2—O1—C1	−165.5 (5)	Co2—O6—C9—C10	−167.4 (2)
N4—Co2—O1—C1	12.5 (5)	C16—C10—C9—O5	−175.1 (4)
O7^ii^—Co2—O1—C1	102.7 (5)	C11—C10—C9—O5	7.2 (6)
O5—Co1—O2—C1	−10.1 (4)	C16—C10—C9—O6	5.2 (6)
O4^i^—Co1—O2—C1	174.5 (3)	C11—C10—C9—O6	−172.5 (3)
N1—Co1—O2—C1	81.4 (3)	C16—C10—C11—C12	−1.8 (6)
N2—Co1—O2—C1	−101.3 (3)	C9—C10—C11—C12	175.9 (3)
O3^i^—Co1—O2—C1	170.2 (3)	C14—C12—C11—C10	1.0 (6)
O5—Co1—N2—C26	52.8 (3)	C13—C12—C11—C10	−178.1 (3)
O2—Co1—N2—C26	164.4 (3)	Co1—O2—C1—O1	−6.7 (6)
O4^i^—Co1—N2—C26	−39.8 (3)	Co1—O2—C1—C2	175.5 (2)
O3^i^—Co1—N2—C26	−100.2 (3)	Co2—O1—C1—O2	84.0 (6)
O5—Co1—N2—C22	−129.2 (3)	Co2—O1—C1—C2	−98.2 (5)
O2—Co1—N2—C22	−17.6 (3)	C11—C10—C16—C15	0.7 (6)
O4^i^—Co1—N2—C22	138.2 (3)	C9—C10—C16—C15	−177.0 (4)
O3^i^—Co1—N2—C22	77.8 (3)	O2—C1—C2—C8	3.3 (5)
O5—Co1—N1—C17	164.0 (4)	O1—C1—C2—C8	−174.7 (4)
O2—Co1—N1—C17	52.3 (4)	O2—C1—C2—C3	−176.9 (3)
O4^i^—Co1—N1—C17	−103.4 (4)	O1—C1—C2—C3	5.2 (5)
O3^i^—Co1—N1—C17	−43.1 (4)	Co2^i^—O7—C13—O8	−0.4 (4)
O5—Co1—N1—C21	−18.8 (4)	Co2^i^—O7—C13—C12	178.8 (3)
O2—Co1—N1—C21	−130.4 (4)	Co2^i^—O8—C13—O7	0.4 (4)
O4^i^—Co1—N1—C21	73.9 (4)	Co2^i^—O8—C13—C12	−178.8 (3)
O3^i^—Co1—N1—C21	134.2 (4)	C14—C12—C13—O7	−11.9 (6)
O1—Co2—N4—C32	76.4 (4)	C11—C12—C13—O7	167.1 (4)
O6—Co2—N4—C32	−167.3 (4)	C14—C12—C13—O8	167.3 (4)
O8^ii^—Co2—N4—C32	−16.8 (4)	C11—C12—C13—O8	−13.6 (6)
O7^ii^—Co2—N4—C32	−75.9 (4)	C3—C2—C8—C7	−2.9 (6)
O1—Co2—N4—C36	−103.6 (3)	C1—C2—C8—C7	177.0 (4)
O6—Co2—N4—C36	12.7 (3)	C6—C7—C8—C2	1.4 (6)
O8^ii^—Co2—N4—C36	163.2 (3)	C8—C7—C6—C4	0.9 (6)
O7^ii^—Co2—N4—C36	104.2 (3)	C3—C4—C6—C7	−1.8 (6)
O1—Co2—N3—C27	179.2 (3)	C5—C4—C6—C7	175.5 (4)
O6—Co2—N3—C27	62.8 (3)	C6—C4—C3—C2	0.3 (6)
O8^ii^—Co2—N3—C27	−87.6 (3)	C5—C4—C3—C2	−177.0 (3)
O7^ii^—Co2—N3—C27	−28.6 (3)	C8—C2—C3—C4	2.0 (5)
O1—Co2—N3—C31	1.1 (4)	C1—C2—C3—C4	−177.8 (3)
O6—Co2—N3—C31	−115.3 (4)	C11—C12—C14—C15	1.0 (6)
O8^ii^—Co2—N3—C31	94.3 (4)	C13—C12—C14—C15	−179.9 (4)
O7^ii^—Co2—N3—C31	153.3 (4)	C12—C14—C15—C16	−2.2 (7)
C26—N2—C22—C23	−1.2 (6)	C10—C16—C15—C14	1.3 (7)
Co1—N2—C22—C23	−179.3 (3)	Co1^ii^—O3—C5—O4	−0.1 (4)
N2—C22—C23—C24	0.7 (7)	Co1^ii^—O3—C5—C4	−178.6 (3)
C22—N2—C26—C25	0.6 (6)	Co1^ii^—O4—C5—O3	0.1 (4)
Co1—N2—C26—C25	178.7 (3)	Co1^ii^—O4—C5—C4	178.6 (3)
C24—C25—C26—N2	0.5 (7)	C3—C4—C5—O3	−173.6 (3)
C32—N4—C36—C35	0.7 (7)	C6—C4—C5—O3	9.1 (6)
Co2—N4—C36—C35	−179.3 (4)	C3—C4—C5—O4	7.9 (6)
C22—C23—C24—C25	0.5 (7)	C6—C4—C5—O4	−169.4 (4)
C26—C25—C24—C23	−1.0 (7)	C31—N3—C27—C28	0.9 (7)
C36—N4—C32—C33	−0.2 (7)	Co2—N3—C27—C28	−177.3 (4)
Co2—N4—C32—C33	179.8 (4)	C27—N3—C31—C30	−0.8 (7)
C21—N1—C17—C18	−0.9 (8)	Co2—N3—C31—C30	177.3 (4)
Co1—N1—C17—C18	176.6 (4)	C28—C29—C30—C31	−0.1 (9)
C17—N1—C21—C20	1.1 (7)	N3—C31—C30—C29	0.5 (9)
Co1—N1—C21—C20	−176.3 (4)	C30—C29—C28—C27	0.2 (9)
N1—C17—C18—C19	0.4 (9)	N3—C27—C28—C29	−0.7 (9)
C35—C34—C33—C32	0.1 (9)		

Symmetry codes: (i) *x*, *y*−1, *z*; (ii) *x*, *y*+1, *z*.

### Hydrogen-bond geometry (Å, º)

**Table:**

*D*—H···*A*	*D*—H	H···*A*	*D*···*A*	*D*—H···*A*
C3—H3···O8^ii^	0.93	2.58	3.494 (5)	167
C11—H11···O4^i^	0.93	2.54	3.461 (5)	171
C19—H19···O8^iii^	0.93	2.60	3.348 (7)	138
C23—H23···O3^iv^	0.93	2.53	3.213 (5)	130
C28—H28···O7^v^	0.93	2.55	3.313 (6)	139

Symmetry codes: (i) *x*, *y*−1, *z*; (ii) *x*, *y*+1, *z*; (iii) −*x*+1, −*y*, −*z*+2; (iv) −*x*+1/2, −*y*+3/2, −*z*+2; (v) −*x*+1, *y*+1, −*z*+3/2.

## References

[bb1] Abourahma, H., Bodwell, G. J., Lu, J., Moulton, B., Pottie, I. R., Walsh, R. B. & Zaworotko, M. J. (2003). *Cryst. Growth Des.* **4**, 513–519.

[bb2] Bruker (2003). *SMART* and *SAINT* Bruker AXS Inc., Madison, Wisconsin, USA.

[bb3] Chen, S. C., Yu, R. M., Zhao, Z. G., Chen, S. M., Zhang, Q. S., Wu, X. Y., Wang, F. & Lu, C. Z. (2010). *Cryst. Growth Des.* **10**, 1155–1160.

[bb4] Farrugia, L. J. (1997). *J. Appl. Cryst.* **30**, 565.

[bb5] Jung, S. S., Dongmok, W., Hyoyoung, L., Sung, I. J., Jinho, O., Young, J. J. & Kimoon, K. (2000). *Nature*, **404**, 982–986.

[bb6] Rosi, N. L., Eckert, J., Eddaoudi, M., Vodak, D. T., Kim, J., O’Keeffe, M. & Yaghi, O. M. (2003). *Science*, **300**, 1127–1129. 10.1126/science.1083440 12750515

[bb7] Sheldrick, G. M. (1996). *SADABS* University of Göttingen, Germany.

[bb8] Sheldrick, G. M. (2008). *Acta Cryst.* A**64**, 112–122. 10.1107/S0108767307043930 18156677

[bb9] Yan, C. W., Li, Y. T. & Liao, D. Z. (1996). *Chin. J. Appl. Chem.* **3**, 60–64.

